# Incidental Learning: A Systematic Review of Its Effect on Episodic Memory Performance in Older Age

**DOI:** 10.3389/fnagi.2019.00173

**Published:** 2019-07-17

**Authors:** Carole C. Wagnon, Katharina Wehrmann, Stefan Klöppel, Jessica Peter

**Affiliations:** University Hospital of Old Age Psychiatry and Psychotherapy, University of Bern, Bern, Switzerland

**Keywords:** episodic memory, incidental learning, intentional learning, neural mechanisms, systematic review, aging

## Abstract

Episodic memory is the capacity to encode, store, and retrieve information of specific past events. Several studies have shown that the decline in episodic memory accompanies aging, but most of these studies assessed memory performance through intentional learning. In this approach, the individuals deliberately acquire knowledge. Yet, another method to evaluate episodic memory performance–receiving less attention by the research community–is incidental learning. Here, participants do not explicitly intent to learn. Incidental learning becomes increasingly important over the lifespan, since people spend less time in institutions where intentional learning is required (e.g., school, university, or at work). Yet, we know little how incidental learning impacts episodic memory performance in advanced age. Likewise, the neural mechanisms underlying incidental learning in older age remain largely unknown. Thus, the immediate goal of this review was to summarize the existing literature on how incidental learning changes with age and how neural mechanisms map onto these age-related changes. We considered behavioral as well as neuroimaging studies using incidental learning paradigms (alone or in combination with intentional learning) to assess episodic memory performance in elderly adults. We conducted a systematic literature search on the Medline/PubMed, Cochrane, and OVID SP databases and searched the reference lists of articles. The search yielded 245 studies, of which 34 concerned incidental learning and episodic memory in older adults. In sum, these studies suggest that aging particularly affects episodic memory after incidental learning for cognitively demanding tasks. Monitoring deficits in older adults might account for these findings since cognitively demanding tasks need increased attentional resources. On a neuronal level, dysregulation of the default-mode-network mirrors monitoring deficits, with an attempt to compensate through increased frontal activity. Future (neuroimaging) studies should systematically evaluate retrieval tasks with diverging cognitive load and consider the influence of attention and executive functions in more detail.

## Introduction

Whether we remember an episode or not depends on a set of mental processes that occur during encoding of this episode, its consolidation and its subsequent retrieval. The capacity to encode, store and retrieve information of personally experienced events is called episodic memory (Tulving, 2016). Episodic memory is essential for daily life and many studies have shown that its performance declines with advancing age (Shing et al., 2010; Nyberg et al., 2012). In the clinical context (e.g., in a memory clinic), episodic memory performance is typically tested by prompting participants to learn (i.e., encode) and retrieve a list of words (Rabin et al., 2005). In these tasks, older adults perform worse during the cognitive demanding *free recall* of words; that is, retrieval without cues (Rhodes et al., 2019). On the contrary, they perform better during the cognitively less demanding *cued recall* or *recognition*; that is, when they receive phonemic (i.e., first letter of the word) or semantic (i.e., the category) cues in case of cued recall or when they perform old/new memory judgements in case of recognition (Rhodes et al., 2019).

**Figure 1:**
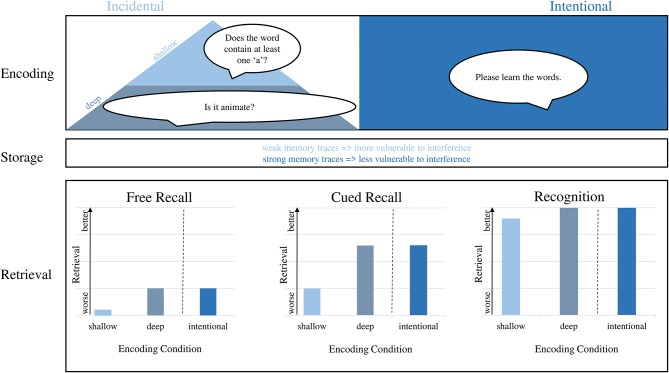
The influence of incidental or intentional encoding on the three stages of episodic memory. During encoding, both deep incidental as well as intentional learning lead to a strong memory trace. During storage, weak memory traces following shallow encoding are more vulnerable to interference than strong memory traces after deep encoding. The disadvantage of shallow incidental encoding on later memory performance is mostly visible during free and cued recall and is almost eliminated during recognition.

The initial encoding of information—be it a word list or other–happens either *intentionally* or *incidentally* (Figure 1). During intentional encoding, participants are instructed to memorize and deliberately direct attention to the stimuli (Ferr et al., 2015). During incidental learning, on the other hand, participants are not aware of the learning situation (i.e., they do not receive the instruction to memorize; McLaughlin, 1965). Their attention is directed to the stimuli because of another task (e.g., categorizing words according to certain criteria) and they encode stimuli “along the way” without the specific intention to do so (Zhou et al., 2012). Although incidental learning becomes increasingly relevant during aging–as people spent less time in institutions where intentional learning is required–most of episodic memory studies in older age focus on intentional learning. Thus, the influence of incidental learning on episodic memory performance in older age remains largely unknown.

One important theory for incidental learning is the *level of processing framework* (Craik and Lockhart, 1972): It postulates that deep (i.e., semantic) compared to shallow (i.e., perceptual) encoding benefits later retrieval (Galli, 2014) and that the retrieval can be further facilitated by using emotional content (either positive or negative; Ferr et al., 2015). In older adults, however, the facilitating effect of deep compared to shallow encoding is under debate. The *processing deficit hypothesis* states that cognitive processing resources are limited in older age and thus, older adults fail to use deeper encoding (Eysenck, 1974). Consequently, their memory performance is worse than in younger adults. On the contrary, the *production deficiency hypothesis* states that older adults are less likely to self-initiate deep encoding (Mitchell and Perlmutter, 1986) but if they are told to use it, they perform comparable to younger adults (Light, 1991).

Irrespective of intentional or unintentional encoding, the interaction between the medial temporal lobe and the prefrontal cortex is crucial for later memory retrieval (Simons and Spiers, 2003)—at least at younger age. In the medial temporal lobe, the hippocampus and its surrounding areas are particularly important for memory consolidation and later retrieval (Simons and Spiers, 2003). The perirhinal cortex is specifically engaged in item encoding (that is, the *what* information; Davachi, 2006) and the right parahippocampal cortex is central for source and associative encoding (that is, contextual details like *where, when*, and *how*; Wheeler and Ploran, 2008). In the prefrontal cortex, the left dorsolateral part coordinates and controls the storing of brain activity patterns via monitoring and verification (Simons and Spiers, 2003), while the right prefrontal cortex is engaged during retrieval processes (Tulving, 2002). Additionally, the left inferior frontal gyrus, the left anterior prefrontal cortex, and the bilateral posterior middle frontal gyrus are active during demanding retrieval task (Wheeler and Ploran, 2008). These regions might provide additional resources to overcome task difficulty and thus, increased activity in these areas may represent compensation in older adults.

In general, aging is accompanied by functional alterations in the brain: Spreading activation (Cabeza, 2002), decreased activity in the medial temporal lobe (Reuter-Lorenz and Park, 2010), and default mode network (DMN) dysregulations (Grady et al., 2010). The DMN is a network of brain regions, which is typically inhibited during cognitive tasks and active during rest as well as mind wandering (Damoiseaux et al., 2008). In older adults, however, the DMN seems to be active also during cognitive tasks (Grady et al., 2010). Because the DMN usually inhibits regions related to attention and control (Broyd et al., 2009), a dysregulation causes higher vulnerability to distractors with a negative effect on memory performance (Lustig et al., 2010). The medial temporal lobe is also strongly affected by age-related alterations (e.g., hippocampal atrophy; Reuter-Lorenz and Park, 2010; Adler et al., 2018). Because of its crucial role for memory encoding, consolidation, and retrieval, these alterations strongly affect memory processing (Simons and Spiers, 2003; Davachi, 2006). For spreading activation, there are different theories: In view of the *dedifferentiation* theory, spreading activation is due to loss of specificity of neural representations in older adults (Baltes et al., 1980). In the *increased noise* theory, dedifferentiation is caused by alternations in neuronal transmission by dopaminergic decline leading to a less distinct neuronal representation (Bäckman et al., 2006). In the *hemispheric asymmetry reduction in older adults* (HAROLD) model, spreading activation is a compensatory mechanism. This theory proposes that older adults tend to show less left lateralized prefrontal activity than younger adults do, in order to meet task demands (Cabeza, 2002). Likewise, prefrontal over-activation is part of the *posterior–anterior shift in aging* (PASA) model, which states that under-activation in posterior regions (i.e., the medial temporal lobe) is typically accompanied by prefrontal over-activation to aid performance (Davis et al., 2008). In a broader sense, the *compensation-related utilization of neural circuits hypothesis* (CRUNCH) states that older adults engage more neuronal circuits than younger adults do, in order to compensate their declining neuronal efficiency–especially for tasks requiring more effort and attention (Reuter-Lorenz and Cappell, 2008). A theory combining dedifferentiation, recruitment of alternative neuronal regions (i.e., prefrontal regions), decreased activation in the medial temporal lobe and DMN dysregulation is the *scaffolding theory of aging and cognition* (STAC) (Reuter-Lorenz and Park, 2010). STAC postulates that these processes are adaptations of the brain to different neuronal age-related challenges like amyloid deposition, atrophy, white matter deterioration and dopamine receptor depletion (Park and Reuter-Lorenz, 2009).

Even though all these theories try to relate functional brain alterations to cognitive performance changes in older adults, none of them provides a specific statement for memory performance. It might be that age-related alterations like decreased activity in the medial temporal lobe as well as dysregulation of the DMN lead to an impaired episodic memory performance, while spreading activation, particularly in the prefrontal cortex, might be beneficial (since more attentional resources are available). Yet, these assumptions still need verification.

Thus, the goals of this review were to survey the literature on how incidental learning changes with advancing age as well as which neural correlates underlie incidental learning in elderly adults.

## Methods

For the present systematic review, we followed the Preferred Reporting Items for Systematic Reviews and Meta-Analysis (PRISMA) guidelines (Moher et al., 2009). We systematically searched for published studies in English with no date restriction across the following databases: Cochrane Central Register of Controlled Trials, MEDLINE, Books@Ovid, Ovid Journals, PsycARTICLES, Ovid MEDLINE(R), Epub, PsycINFO, and PSYNDEXplus. We used the following search terms: “incidental learning AND episodic memory AND older adults NOT child NOT animal NOT Alzheimer NOT MCI.” For the neuroimaging part, we applied an additional literature search with the following search terms: “brain AND age AND [incidental/level of processing/categorical decision] NOT children NOT patients NOT working memory NOT Alzheimer NOT MCI NOT animals NOT dementia NOT Parkinson NOT infants NOT incidental findings NOT alcohol NOT stimulation NOT motor NOT STROOP NOT depression NOT syndrome NOT smoking NOT stress NOT diabetes NOT tinnitus NOT eye-tracking.”

To be eligible for inclusion, studies needed to: (1) investigate incidental learning (preferably, but not necessarily, in conjunction with intentional learning) in cross-sectional or longitudinal designs, (2) focus on episodic memory, (3) include older adults or compare older adults' performance to that of younger participants. To reduce the risk of bias, two authors (CW and KW) independently screened abstracts and titles and analyzed studies that met inclusion criteria, as suggested by the PRISMA guidelines. We also screened the reference lists of included studies to identify any additional studies.

**Figure 2:**
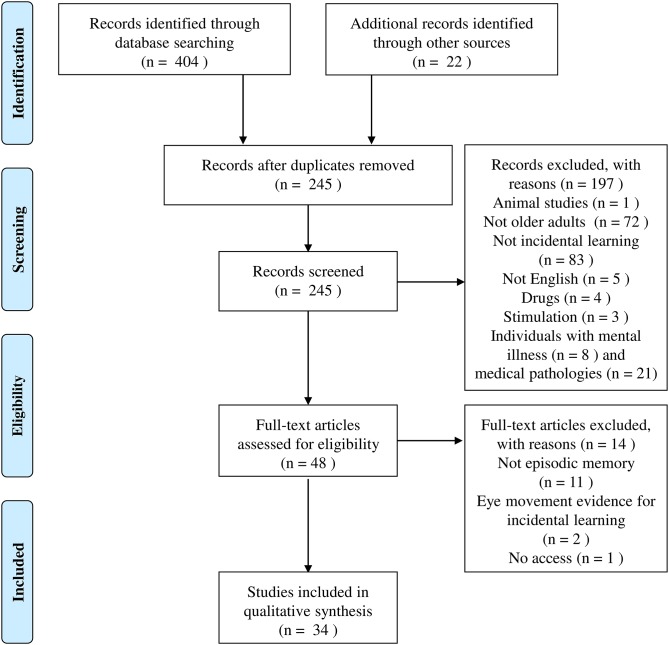
Flow chart of the identification and inclusion of studies in the current review.

The search yielded 726 studies, of which 33 met criteria for the final review. Figure 2 reports the four phases of the selection process (identification, screening, eligibility, and inclusion—as suggested by PRISMA).

## Results

**Figure 3:**
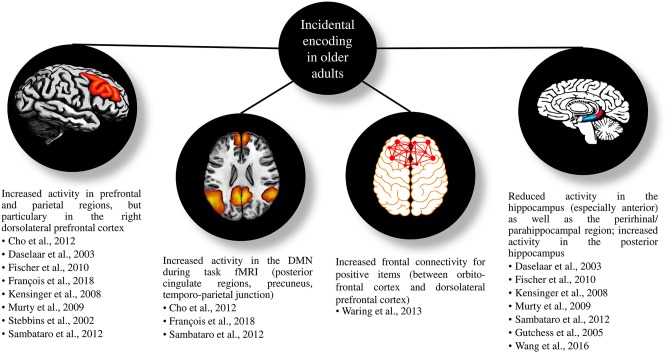
Figure of the most important functional magnetic resonance imaging results of the reviewed studies when applying incidental encoding in older compared to younger adults. DMN, default mode network; fMRI, functional Magnetic Resonance Imaging.

Table 1 gives an overview on the included studies (see also Table 2 for a summary on the behavioral findings). The majority of these studies found comparable or decreased episodic memory performance in older adults compared to younger participants, while only one study evidenced superior performance. Age-related changes were more evident in retrieval tasks with high cognitive load (i.e., free recall) than in less demanding retrieval tasks (i.e., recognition). For less demanding tasks, older adults showed higher false alarm rates compared to younger adults but hit rates and reaction times were comparable. Fifteen studies examined age-related changes in neural correlates of incidental learning and observed less activity in hippocampal regions as well as more activity in right frontal regions. Likewise, regions related to the DMN tended to be more active in older adults compared to younger adults (see Figure 3 for a summary). In the following, we will describe the reviewed studies in more detail. We will first concentrate on studies, which applied incidental learning only, afterwards review studies that applied both incidental and intentional learning and finally, we will focus on studies that investigated the neural correlates of incidental learning in older age.

**Table 1:** Overview of all included studies.

	**Methods**						
**References**	**Participants**	**Condition**	**Imaging**	**Stimuli**	**Encoding type**	**Retrieval**	**Results**
Eysenck, 1974	*N* = 100 (*n* = 50 o, *n* = 50 y)	Incidental + intentional	No	Words	Deep and shallow	Free recall	y = o for shallow items y > o for deep items y > o for intentional condition
Mason, 1979	*N* = 498 (*n* = 190 o, *n* = 136 mo, *n* = 172 y)	Incidental + intentional	No	Words	Deep and shallow	Free recall and recognition	o = y for shallow items y > o for deep items y > o for intentional condition
Duchek, 1984	*N* = 64 (*n* = 32 o, *n* = 32 y)	Incidental	No	Words	Deep and shallow	Cued recall	y > o for deep and shallow condition
Mitchell and Perlmutter, 1986	*N* = 64 (*n* = 32 o, *n* = 32 y)	Incidental + intentional	No	Words	Deep and shallow	Free recall and recognition	y = o for shallow and deep condition y > o for intentional condition
Mitchell, 1989	*N* = 96 (*n* = 48 y, *n* = 48 o)	Incidental	No	Line drawings	Picture-naming	Free Recall and recognition	y > o
Stebbins et al., 2002	*N* = 30 (*n* = 15 y, *n* = 15 o)	Incidental	fMRI	Words	Deep and shallow	None	y > o (activity in the left superior and middle frontal gyrus)
Daselaar et al., 2003	*N* = 60 (*n* = 20 y, *n* = 40 o)	Incidental	fMRI	Words	Emotional and shallow	Recognition	y > o (activity in the perirhinal/parahippocampal region) y < o (activity in the right prefrontal cortex)
Aine et al., 2005	*N* = 20 (*n* = 10 y, *n* = 10 o)	Incidental	MEG	Words	Deep	Recognition	y = o y = o (time courses in the prefrontal cortex) y < o (higher amplitudes in the prefrontal cortex)
Gutchess et al., 2005	*N* = 27 (*n* = 14 y, *n* = 13 o)	Incidental	fMRI	Pictures (scenes)	Shallow	Recognition	y = o y > o (activity in both parahippocampi) y < o (activity in middle frontal cortex and stronger negative connectivity between parahippocampal and inferior frontal cortex)
Troyer et al., 2006	*N* = 104 (*n* = 40 y, *n* = 64 o)	Incidental + intentional	No	Names and faces	Deep and shallow	Free recall and recognition	y = o for incidental y > o for intentional
Kensinger and Schacter, 2008	*N* = 37 (*n* = 17 y, *n* = 20 o)	Incidental	fMRI	Pictures (objects)	Deep	Recognition	y > o (negative and neutral items; proportion of correctly recognized “same” items) y > o (activity in the hippocampus and the parahippocampal gyrus) y < o (activity in the medial, middle and inferior frontal gyrus, the middle temporal gyrus, and anterior cingulate gyrus)
Murty et al., 2009	*N* = 60 (30 y, *n* = 30 o)	Incidental	fMRI	Emotional scenes	Deep	Recognition	y > o (accuracy and reaction times) y > o (activity in hippocampus and amygdala) y < o (activity in ventral visual stream, prefrontal, and parietal cortex)
Naveh-Benjamin et al., 2009	*N* = 47 (*n* = 24 y, *n* = 23 o) *N* = 84 (*n* = 42 y, *n* = 42 o)	Incidental + intentional	No	Names and faces	Face-name matching or association	Recognition	y = o for intentional except for associations (here, y > o) y > o for incidental
Fischer et al., 2010	*N* = 45 (*n* = 24 y, *n* = 21 o)	Incidental	fMRI	Emotional faces	Emotional	Recognition	y = o (hits) y < o (false alarm and discrimination) y > o for fearful vs. neutral faces (activity in right amygdala and bilateral hippocampus) y < o for fearful vs. neutral faces (activity in left insular cortex and right superior frontal gyrus)
Plancher et al., 2010	*N* = 160 (*n* = 82 y, *n* = 78 o)	Incidental + intentional	No	Urban environment in VR	Driving in VR	Free recall and recognition	y < o for incidental “what” details y = o for intentional “what” details and for incidental “when” details y > o for ‘where' details in both conditions
Cho et al., 2012	*N* = 63 (*n* = 40 y, *n* = 23 o)	Incidental	fMRI	Words (auditory)	Deep	None	y > o (inferior frontal gyrus and middle temporal gyrus) y < o (ventromedial prefrontal cortex, right middle, and inferior frontal gyrus, bilateral precuneus, left middle, and superior temporal gyrus, bilateral parahippocampus, and bilateral posterior cingulate cortex)
Sambataro et al., 2012	*N* = 44 (*n* = 22 y, *n* = 22 o)	Incidental	fMRI	Pictures (scenes)	Shallow	Recognition	y > o (left hippocampus) y < o (bilateral prefrontal cortex, precuneus, temporo-parietal junction, and posterior cingulate regions)
Waring et al., 2013	*N* = 37 (*n* = 19 y, *n* = 18 o)	Incidental	fMRI	Emotional scenes	Emotional	Recognition	y > o y < o (stronger connectivity in frontal regions and from frontal regions to medial temporal lobe structures)
Greve et al., 2014	*N* = 48 (*n* = 24 y, *n* = 24 o)	Incidental + intentional	sMRI	Pictures (objects)	Shallow	Recognition	y > o
Martins et al., 2014	*N* = 42 (*n* = 28 y, *N* = 14 o)	Incidental	fMRI	Words	Deep and shallow	None	y > o for deep vs. shallow encoding (left prefrontal cortex, left posterior cingulate cortex, left precuneus y < o for shallow vs. deep encoding (left posterior cingulate cortex)
Kalenzaga et al., 2015	*N* = 35 (*n* = 19 y, *n* = 16 o)	Incidental	fMRI	Sentences	Self-referential and imagery	Free recall	y = o for item memory y > o for source memory y < o (activity in fronto-parietal network)
Carr et al., 2015	*N* = 71 (*n* = 47 y, *n* = 24 o)	Incidental	No	Faces	Similarity and distinctiveness	Recognition	y > o for distinctiveness y = o for similarity
Lindner et al., 2015	*N* = 55 (*n* = 36 y, *n* = 19 o); *N* = 66 (*n* = 26 y, *n* = 30 o); *N* = 43 (*n* = 24 y, *n* = 19 o)	Incidental + intentional	No	Sentences	Source and destination	Recognition	y = o for source and destination in both conditions
Ramanoël et al., 2015	*N* = 24 (*n* = 12 y, *n* = 12 o)	Incidental	fMRI	Pictures (scenes)	Shallow	None	y < o (right middle frontal gyrus, right inferior parietal lobule, left inferior parietal lobule, and left middle temporal gyrus)
Saverino et al., 2016	*N* = 34 (*n* = 16 y, *n* = 18 o)	Incidental	fMRI	Pictures (house/objects)	Shallow and deep	Recognition	y > o for association y = o for categorization y = o (parahippocampal gyrus, inferior parietal lobe) y > o (precentral gyrus, inferior temporal gyrus, posterior cingulate gyrus, precuneus)
Wang and Giovanello, 2016	*N* = 52 (*n* = 29 y, *n* = 23 o)	Incidental + intentional	fMRI	Sentences	Reading	Recognition	y = o for incidental y = o for intentional y = o (hippocampus and perirhinal cortex)
Fu et al., 2017	*N* = 46 (*n* = 23 y, *n* = 23 o)	Incidental	No	Words	Deep and shallow	Recognition	y > o for shallow y = o for deep
Kontaxopoulou et al., 2017	*N* = 47 (*n* = 27 y, *n* = 20 o)	Incidental + intentional	No	Computer-generated items (i.e., speed limit signs), words, and geometric figures	Computer-generated driving task, verbal learning, and visuospatial memory	Free Recall and recognition	y > o for incidental free recall, intentional visuospatial free recall, and incidental recognition y = o for intentional verbal free recall, intentional verbal recognition, and incidental visuospatial recognition
François et al., 2018	*N* = 39 (*n* = 20 y, *n* = 19 o)	Incidental	fMRI	Drawings	Deep	Recognition	y > o except for reaction times or new items (here, y = o) y < o (right frontal areas and regions associated with the DMN)
Hämmerer et al., 2018	*N* = 50 (*n* = 28 y, *n* = 22 o)	Incidental	sMRI	Pictures (scenes)	Shallow	Recognition	y = o (hits, false alarms) y = o (volume of the locus coeruleus)
Hennessee et al., 2018	*N* = 66 (*n* = 33 y, *n* = 33 o)	Incidental +intentional	No	Words with color and point-value	Binding of color and point-value or word learning	Recognition	y > o for intentional y = o for incidental
Lugtmeijer et al., 2019	*N* = 59 (*n* = 30 y, *n* = 29 o)	Incidental	No	Pictures (objects)	Object-location binding	Recognition	y > o for location
Meade et al., 2018	*N* = 144 (*n* = 72 y, *n* = 72 o)	Incidental	No	Words (objects)	Drawing, writing, or listing characteristics of the objects	Free recall and recognition	y > o except for encoding via drawing (here, y = o for hit rate)

*y, young; mo, middle-old; o, old; fMRI, functional Magnetic Resonance Imaging; sMRI, structural Magnetic Resonance Imaging; MEG, Magnetoencephalography; VR, virtual reality; DMN, default mode network*.

**Table 2:** Summary of behavioral findings when comparing episodic memory performance following incidental or intentional learning in groups of healthy young and elderly adults.

**Performance measure**		**Comparison of episodic memory performance**		
		**Young > old**	**Young = old**	**Young < old**
Free recall	Incidental	Eysenck (deep) Mason (deep) Mitchell Plancher Kontaxopoulou	Eysenck (shallow) Mason (shallow) Mitchell & Perlmutter (shallow) Mitchell & Perlmutter (deep) Plancher	Plancher
	Intentional	Eysenck Mason Mitchell & Perlmutter Plancher Kontaxopoulou	Plancher Kontaxopoulou	
Cued recall	Incidental	Duchek (shallow + deep)		
Recognition (hit rate)	Incidental	Mason (deep) Naveh-Benjamin Carr Saverino Fu (shallow) Francois Waring Greve Meade Kontaxopoulou Murty Kensinger	Mitchell Mitchell & Perlmutter Daselaar Sambatoro Carr Kalenzaga Lindner Fu (deep) Lugtmeijer Hennessee Hämmerer Gutchess Aine Troyer Wang Meade Kontaxopoulou Fischer Kensinger	
	Intentional	Mitchell & Perlmutter Hennessee Troyer	Naveh-Benjamin Lindner Wang Kontaxopoulou	
Recognition (false alarm rate)	Incidental	Mitchell Carr Saverino Francois Gutchess Fischer	Lugtmeijer Hämmerer	
	Intentional		Hennessee	
Recognition (reaction times)	Incidental		Daselaar Francois Aine	Murty

*Please note that some studies applied several experiments (e.g., Plancher et al., 2010). For false alarms, lower false alarm rates indicate better performance*.

Duchek (1984) tested incidental learning in a cued recall task after a semantic (e.g., “is it a type of bird?”) or rhyme (e.g., “does it rhyme with care?”) categorization task. Older adults remembered fewer items than younger adults did and were overall slower in their reactions. Younger participants remembered significantly more yes-responses than no-responses and were superior in recall of semantically encoded words.

Kalenzaga et al. (2015) found no significant age difference in free recall of autobiographical memories. In their task, participants had to fill in sentences with one missing word and think about an experience they made on this topic.

Several studies investigated the effect of deep vs. shallow encoding on free recall of words but yielded contradicting results. Eysenck (1974) and Mason (1979) found comparable memory performance in both age groups after shallow encoding but inferior retrieval in older adults following deep encoding. In contrast, Mitchell and Perlmutter (1986) as well as Fu et al. (2017) found elderly adults to benefit from deep encoding with comparable performance during retrieval or recognition. However, the effect sizes in these studies were small (*d* = 0.25–0.34), with the highest effect size observed by Eysenck (1974). All of these studies additionally applied intentional encoding and constantly found younger adults outperforming older adults at retrieval.

Daselaar et al. (2003) applied encoding and recognition of words rated according to their pleasantness (i.e., is the word pleasant or unpleasant). During recognition, the participants had to decide if a given word was old or new. The authors did not find any significant difference between young and elderly individuals regarding hit rate or reaction times.

Similarly, Sambataro et al. (2012) found no significant age difference in recognition accuracy. In their task, the participants had to decide if a given image represented an indoor or an outdoor scene and in a subsequent recognition task, they had to recognize if the images were old or new. On the contrary, Murty et al. (2009) found superior performance of younger adults compared to older adults for accuracy and reaction times during a similar task (i.e., recognition of indoor and outdoor scenes).

Mitchell (1989) examined the effect of incidental learning on subsequent free recall and recognition. Participants had to name pictures, which appeared on a projection screen and, after a short delay, recalled the names of the pictures in writing and performed an old/new recognition task. Younger participants freely recalled more pictures and had a lower false alarm rate during recognition compared to older participants, but both groups performed comparable for hit rates.

Carr et al. (2015) led participants decide if several faces were distinct or similar to a given face. In the subsequent recognition task, the group of younger participants outperformed the group of older adults only for faces studied in the distinctness task but not for faces studied in the similarity task. In a similar approach, Fischer et al. (2010) led participants decide if a face was fearful or neutral. They found no age effect for hits in the subsequent recognition task, but younger adults outperformed older adults with lower false alarm rates.

François et al. (2018) led participants decide if a given line drawing would fit into a shoebox or not. In the subsequent recognition task (remember/know/new), they found no significant differences in reaction times between both groups. However, they observed lower hit rates for remember items in older adults compared to younger adults and an increased false alarm rate for both remember and know items. No significant differences were found for items declared as new (neither hit rate nor false alarm rate). (Kensinger and Schacter, 2008) performed a similar study by asking participants if several objects would fit in a file cabinet drawer or not. Older adults showed lower hit rates, particularly for negative and neutral items. However, they recognized positive items comparable to younger adults. Thus, older adults remember the positive material better, indicating a well-preserved positivity effect in this population (Carstensen and Mikels, 2005).

Comparable to Daselaar et al. (2003), Sambataro et al. (2012), Kalenzaga et al. (2015), and Lugtmeijer et al. (2019) found no age-related difference in recognition performance after incidental learning. Similarly, Aine et al. (2005), Gutchess et al. (2005), and Hämmerer et al. (2018) found no evidence of a recognition deficit in older adults. In contrast, Waring et al. (2013), Greve et al. (2014), and Meade et al. (2018) evinced impaired recognition performance in older adults after both incidental and intentional encoding. Meade et al. (2018) compared the effect of different encoding strategies on word retrieval. They asked participants to draw the to-be-remembered words, to create a mental image of the object, or to write down as many characteristics of the object as possible. Only drawing of the object increased performance in older adults in a way that they performed similarly to younger adults during recognition.

Saverino et al. (2016) tested item and associative encoding under incidental conditions. For item encoding, participants indicated if the style of a given house on a picture was modern or traditional. During associative encoding, they decided if “based on the style of an object, would it be likely to be found in the house.” Later on, they administered an old/new recognition task. The authors found no significant age differences for item recognition but older adults performed significantly worse for associative recognition with a lower number of hits and a higher false alarm rate. This supports an associative memory deficit in older adults, which was also found in a study by Naveh-Benjamin et al. (2009). Here, participants were either asked to remember face-name pairs (i.e., intentional condition) or to rate whether a name fits to a face (i.e., incidental condition). The authors found that younger and older adults had comparable memory performance for faces and names in isolation, irrespective of incidental, or intentional learning. However, an age-related deficit appeared specific to associations under intentional—but not incidental—learning. This was due to a higher false alarm rate during the associative task.

Troyer et al. (2006) conducted two experiments with younger and older adults. They applied three different levels of incidental encoding (physical, phonematic, semantic) to surnames or face-name pairs and administered intentional learning, too. Younger participants outperformed older ones during free recall as well as recognition of intentionally learned names but showed equal performance during free recall and recognition of intentionally learned face-name-pairs. For incidental learning, both groups performed equally well during free recall and recognition of both names and face-name pairs. On the contrary, Wang and Giovanello (2016) found no significant age difference for incidental encoding of word pairs that appeared together during a sentence-reading task.

In a very recent study, Hennessee et al. (2018) asked participants to imagine being in different states of physiological need (e.g., hunger, thirst) as well as being in different locations (e.g., kitchen, forest). Then, they should examine the congruence of an item with the state of need and to rate how much they want to have this item right now. After a delay, they applied an old/new recognition task. The authors found comparable performance for hit rates but older adults showed a significantly higher false alarm rate. In a second experiment, they showed words in different colors, associated with different point-values. They asked the participants to memorize the words but they did not ask them to memorize the color nor the value. Younger adults outperformed older individuals for low value items but not for high value items. The authors found no significant age difference for incidental learning (color).

In a study by Lindner et al. (2015), participants had to listen to sentences and to encode the source (i.e., who said something) or the destination (i.e., to whom was said something); thus they encoded the *where* details. During retrieval, they had to decide whether sentences were spoken by/to a person and if a sentence was old or new. They were either made aware of the upcoming memory test (i.e., intentional learning) or not (i.e., incidental learning). The authors observed no significant differences in recognition performance between both age groups in either learning condition. Similarly, Plancher et al. (2010) were interested in *where* as well as in *what* and *when* details. They asked participants to drive through a virtual town and to pay attention to the surroundings (incidental condition) or to try to remember the itinerary (intentional condition). After a short delay, they first performed a free recall on *what, where*, and *when* details associated with the itinerary and afterwards applied a recognition task in which the participants decided which item among three different ones appeared in the task. They found an increased memory performance among older adults under incidental learning and comparable performance under intentional learning (but only for *what* details). In contrast to Lindner et al. (2015), older adults scored less on recall of *where* information, both under intentional and incidental encoding using a free recall task. For the *when* information, young participants outperformed older ones under intentional—but not incidental—encoding. Likewise, Kontaxopoulou et al. (2017) examined age effects for incidental encoding of *what* details during a driving simulator task. They also applied intentional encoding, by asking participants to learn line drawings in different spatial locations as well as words. In contrast to the results of Plancher et al. (2010), older adults showed worse recall and recognition performance for incidentally learned *what* details. For intentionally learned items, only the free recall of line drawings revealed significant age differences as older males performed worse than younger males did. Taken together, the difficulty of the retrieval task seems to explain age-related differences rather than the content of the remembered details (i.e., *what, where*, or *when* information).

In the following, we will describe neuroimaging studies that applied incidental encoding in young and elderly adults (see Figure 3 for a summary of the findings).

Daselaar et al. (2003), Gutchess et al. (2005), Kensinger and Schacter (2008), Murty et al. (2009), Fischer et al. (2010), and Sambataro et al. (2012) found reduced activity in medial temporal lobe structures as well as stronger activity in frontal regions in older adults, which is in line with the PASA model. According to the model, under-activation in posterior regions in older adults is typically associated with prefrontal over-activation; the latter representing additional resources to overcome cognitive impairment (Davis et al., 2008). Murty et al. (2009) investigated brain activity differences between younger and older adults during encoding and retrieval of indoor and outdoor scenes. They found decreased activity in the hippocampus and the amygdala accompanied by increased activity in frontal and parietal cortices during encoding and retrieval in older adults. Gutchess et al. (2005) tested the contrast between remembered and forgotten items during the encoding of indoor and outdoor scenes. Both age groups showed comparable activity in bilateral inferior frontal regions, regions of the dorsal and ventral stream, and fusiform areas. Older adults exhibited less activity in the parahippocampus (both sides) but more activity in the left middle frontal cortex compared to younger adults. Increased activity in inferior frontal regions was associated with lower parahippocampal as well as higher middle frontal activity in older adults. Frontal connectivity during encoding correlated significantly with later memory performance. Daselaar et al. (2003) observed reduced activity in the perirhinal/parahippocampal cortex during incidental encoding of words in older participants. They further discovered a trend for a reduced lateralization of prefrontal activity in the older group. Activity in the perirhinal/parahippocampal cortex during encoding is crucial for later retrieval (Strange et al., 2002) and reduced activity in these regions in older adults might indicate an encoding deficit (Daselaar et al., 2003). Sambataro et al. (2012) observed decreased activity in the left hippocampus as well as increased activity in bilateral prefrontal cortical regions in older participants compared to younger ones during incidental encoding of scenes. Similarly, Fischer et al. (2010) found decreased hippocampal activity in older adults compared to younger adults during encoding of fearful faces accompanied by decreased activity in the right amygdala as well as increased activity in the left insular cortex and the right superior frontal gyrus. In Kensinger and Schacter (2008), successful encoding was associated with increased activations in the bilateral hippocampus and the bilateral parahippocampus in younger adults, while it was associated with more activity in the bilateral medial, the left middle and the right inferior frontal gyrus, the right middle temporal gyrus, the right insula, and the bilateral anterior cingulate gyrus in older adults. Again, this speaks to the PASA model.

Waring et al. (2013) compared effective brain connectivity during encoding of emotional items and their background and observed stronger connectivity in frontal regions and from frontal regions to medial temporal lobe structures in older adults. These results correspond to the PASA model as well as the CRUNCH model (since the findings were more prominent in difficult tasks).

Wang and Giovanello (2016) observed similar activity in the hippocampus and the perirhinal cortex in both age groups, but the posterior part of the hippocampus was more active during retrieval in older adults. Another study of the same group found that the posterior hippocampus and the posterior medial cortex were stronger functionally connected in older participants (Wang et al., 2010).

Evidence for the HAROLD model provide the studies of Stebbins et al. (2002), Sambataro et al. (2012), Kalenzaga et al. (2015), and François et al. (2018). Kalenzaga et al. (2015) found increased activity in fronto-parietal regions when comparing older adults to younger ones after forming associations between words out of a sentence-filling task and autobiographical memories. Likewise, Stebbins et al. (2002) observed that older adults show less left-lateralized activity during encoding than younger adults did, especially in the superior and middle frontal gyrus. They asked participants to decide if words were abstract or concrete (deep encoding) or if the words were printed in uppercase or lowercase letters (shallow encoding). François et al. (2018) compared brain activity for remember vs. know items after encoding of line drawings. Both age groups showed increased activity during encoding in the right inferior frontal gyrus and the pre-supplementary motor area for remember compared to know items. For remember items, younger adults showed increased bilateral activity in the inferior frontal gyri as well as in the left middle temporal gyrus. Older adults showed increased activity in the left and right precuneus, the right superior temporal gyrus, and the right middle as well as superior frontal gyri. Likewise, Sambataro et al. (2012) observed increased activity in bilateral prefrontal regions in older participants compared to younger ones during incidental encoding of scenes.

Aine et al. (2005) used magnetoencephalography (MEG) to identify time-dependent changes of the magnetic field in the brain during incidental encoding processes. In their study, the participants had to decide if presented objects were larger than a television or not. The authors found similar time-dependent changes in prefrontal regions in both age groups, but elderly adults tended to produce stronger responses than younger adults did.

Stronger activity in the precuneus and the superior temporal gyrus in older adults as observed in a study by François et al. (2018) might be an indicator for a less attenuated DMN as well as an inhibited fronto-parietal network. Likewise, Sambataro et al. (2012) observed that regions related to the DMN were more active during incidental encoding of scenes in older adults compared to younger ones. The authors stated that the increased activity in the DMN reflects additional allocation of attentional resources, which supports the STAC model. Others favored increased activity in the DMN as a sign of dysregulation leading to reduced ability to control attention (Reuter-Lorenz and Park, 2010).

Saverino et al. (2016) found similar activity in the right middle occipital gyrus and the left parahippocampus in both groups during encoding of house pictures. For incidental associative encoding of objects, elderly adults exhibited decreased activity in the inferior frontal gyrus, the left precuneus, the right inferior temporal gyrus, and the left middle as well as the right posterior cingulate cortex. The authors suggested this as a sign for dedifferentiation in older adults, which means that older adults have less distinct neuronal representations for associative encoding, indicating a breakdown of functional specificity (Zelinski and Lewis, 2003). Cho et al. (2012) found similar results when they let participants decide if auditory presented words belong to a certain category or not. Older adults showed broad activation in right frontal regions (middle and inferior frontal gyrus and ventromedial prefrontal cortex) and in the DMN but also in the bilateral parahippocampus. This favors the dedifferentiation theory again, supporting that brain activity in older adults is less lateralized than in younger adults. Likewise, it might indicate that broader activity in older adults represents compensatory mechanisms. Ramanoël et al. (2015) also found more activity in older adults compared to younger adults during the categorization of indoor and outdoor scenes. Again, they found that the DMN is more active in older adults compared to younger adults during task execution.

Martins et al. (2014) assessed brain activity during semantic and phonological decisions (i.e., deep and shallow encoding). The contrast between these two encoding conditions revealed increased left lateralized activation (prefrontal cortex, posterior cingulate cortex, and precuneus) in younger participants. Interestingly, these regions are typically more active in older adults during compensation. Thus, when facing complex tasks (i.e., deep encoding), younger adults show increased activity in brain regions, which are associated with compensational approaches in older adults. Yet, in older adults, the semantic and phonological routes seem to merge into one single pathway. Thus, older adults seem to encode similarly during shallow and deep encoding, leading to better performance after shallow encoding but worse performance after deep encoding.

## Discussion and Perspective

This review revealed several important findings on how incidental learning changes with advancing age as well as how these changes relate to episodic memory performance.

First, episodic memory following incidental learning seems to be more impaired in older adults compared to younger adults in retrieval tasks with high cognitive load (i.e., free recall) compared to less demanding retrieval tasks (i.e., recognition; Figure 1). This is in line with a former meta-analysis, which summarized that age differences following incidental learning are present during free recall, attenuated during cued recall and are eliminated during recognition (Old and Naveh-Benjamin, 2008). There are several explanations for this finding: During free recall, older adults do less often use search strategies spontaneously to enhance retrieval (Lemaire, 2010; Cadar et al., 2018). Even if such strategies are provided, they use them less frequently than younger adults do and, consequently, their recall ability is lower (Lemaire, 2010). Furthermore, they regularly exhibit higher rates of intrusions (Kahana et al., 2005). During recognition, search strategies are not that important, which might explain why only few studies found age-related differences for recognition tasks.

Second, older adults retrieve less during free recall but the depth of processing influences the performance (at least in two out of three studies): Older adults perform comparable after incidental encoding with shallow processing (i.e., when focusing on the appearance of stimuli), while younger participants mostly outperform elderly participants after deep encoding (i.e., when focusing on the meaning of stimuli). These findings might be explained by impaired cognitive control processes in older adults and, thus, loss of attentional resources (Mather and Carstensen, 2005). Cognitive control is the ability to limit attention to goal-relevant information and inhibit, or suppress, irrelevant distraction (Houghton and Tipper, 1996). Deep processing demands directed attention to the task and, therefore, may be more affected by age-related attentional deficits than shallow processing, which demands less attentional resources (Craik and Lockhart, 1972). In sum, the results of this review favor the processing deficit hypothesis over the production deficiency hypothesis but further research might help to provide a definite statement.

Third, in recognition tasks, worse performance in elderly compared to younger participants is more likely for false alarms than for hit rates or reaction times. A higher false alarm rate in older adults also emerges after intentional encoding, which is known as the *false-recognition effect* (Balota et al., 1999): Older adults often intermingle distractor items for “old” items, particularly if they are semantically, phonologically, or orthographically related to previously shown items (Schmid et al., 2010). Since old/new decisions require proper monitoring abilities, a higher false alarm rate indicates a monitoring deficit. Increasing attention toward stimuli does not substantially alter the false-recognition effect (Koutstaal et al., 1999).

Fourth, we found evidence for an associative-memory deficit in older adults after incidental encoding, which tended to be larger after intentional encoding. Deficits in strategic processing, which are not required for incidental encoding seem to be responsible for this finding (Naveh-Benjamin et al., 2009).

When applying both incidental and intentional learning, age effects were more prevalent in free recall as well as recognition performance following intentional learning. There seems to be an influence of stimulus material, at least for the free recall performance: Older adults exhibit worse performance for the recall of *when* and *what* information after intentional—but not incidental—learning. However, they show impaired recall of *where* information for both learning conditions.

In sum, older adults perform inferior to younger adults following intentional learning as well as deep incidental learning, but they perform similar after shallow incidental learning. Tasks with high cognitive load (i.e., free recall) show more age-related impairment than less demanding tasks (i.e., recognition). A monitoring deficit in older adults seems to be responsible since intentional learning as well as deep encoding require more effort and attention (Troyer et al., 2007).

Regarding the neural correlates of incidental learning, we also found a few important findings (see Figure 3 for a summary). However, the interpretation might be limited since only one study corrected for volume differences (Stebbins et al., 2002).

First, most of the studies showed broader activity in older adults than in younger adults, mostly in the right prefrontal cortex. This is in line with the HAROLD model stating that older adults additionally activate right prefrontal areas to meet task demands (Cabeza, 2002). Since the prefrontal cortex is related to attention, increased activity in this area indicates that older participants require more attentional resources (Shing et al., 2010).

Second, the DMN is active during tasks in older adults and thus, inhibits brain areas involved in focusing and directing attention during a task. The dysregulated DMN hinders memory processes, which rely on focused attention (Shing et al., 2010). According to the STAC model, activity in frontal brain regions compensates dysregulation in order to maintain cognitive functioning (Reuter-Lorenz and Park, 2010). Thus, increased activity in the prefrontal cortex, which was continuously found in older adults, may compensate dysregulation in the DMN (Shing et al., 2010).

Third, less activity in the hippocampus during incidental encoding accompanies the broader frontal activity in older adults. This is in line with the PASA model stating that under-activation in the medial temporal cortex may be compensated with over-activity in the prefrontal cortex (Davis et al., 2008). During retrieval, older adults increasingly activate the posterior hippocampus, which might also indicate compensation (Gunning-Dixon et al., 2003).

Fourth, one study provides some indication why shallow encoding is well-preserved in older adults in contrast to deep encoding: Older adults show no significant brain activation difference for shallow compared to deep encoding tasks, while younger participants increase activity for the latter. This might indicate that older adults do not adapt to tasks that are more complex, which is why they show good performance in shallow tasks but worse performance in deep encoding tasks.

In sum, only few studies so far investigated how aging affects incidental learning. These studies found superior performance of younger adults in free recall tasks, particularly after intentional learning. On the contrary, older adults performed similar to younger adults in less cognitively demanding retrieval tasks (i.e., recognition), regardless of intentional or incidental encoding. Monitoring deficits in older adults might account for these findings since cognitively demanding free recall tasks need increased attentional resources. Regarding the neural correlates of incidental learning in older age, even less studies were available. These found broader activity in prefrontal regions, increased activity in the DMN during tasks, and less activity in hippocampal regions in older adults. Dysregulation of the DMN might indicate problems with monitoring, while increased prefrontal activity might signal compensation to account for deficits in attention.

In the future, more studies should systematically manipulate incidental encoding with different depths of processing and subsequently evaluate its effect on retrieval tasks with diverging cognitive load (i.e., free recall vs. recognition). Future studies should also consider the influence of attention and executive functions (i.e., monitoring) in more detail. We additionally suggest including both incidental and intentional encoding in future studies to allow for a direct comparison. More functional neuroimaging studies might foster our understanding of the age impact on the different stages of episodic memory and the contribution of hippocampal subregions. Importantly, these studies should account for age-related brain volume changes. For the DMN, resting-state connectivity might disentangle if increased activity is a sign of dysregulation.

## Data Availability

No datasets were generated or analyzed for this study.

## Author Contributions

JP and SK contributed conception and design of the study. CW and KW performed the statistical analysis. CW wrote the first draft of the manuscript. All authors contributed to manuscript revision, read, and approved the submitted version.

### Conflict of Interest Statement

The authors declare that this study received funding from the Novartis Foundation for medical-biological research. The funder had no role in study design, data collection and analysis, decision to publish, or preparation of the manuscript.
